# Participation in a scientific pre-university program and medical students’ interest in an academic career

**DOI:** 10.1186/s12909-017-0990-4

**Published:** 2017-09-05

**Authors:** Wendy E. de Leng, Karen M. Stegers-Jager, Marise Ph. Born, Maarten A. Frens, Axel P. N. Themmen

**Affiliations:** 1 Institute of Medical Education Research Rotterdam (iMERR), Erasmus MC, PO Box 2040, 3000CA, Rotterdam, The Netherlands; 2 Department of Psychology, Erasmus University Rotterdam, PO Box 1738, 3000DR, Rotterdam, The Netherlands; 3 Department of Neuroscience, Erasmus MC, PO Box 2040, 3000CA, Rotterdam, The Netherlands; 4 Erasmus University College, Erasmus University Rotterdam, Nieuwemarkt 1A, 3011HP, Rotterdam, The Netherlands; 5 Department of Internal Medicine, Erasmus MC, PO Box 2040, 3000CA, Rotterdam, The Netherlands

**Keywords:** Academic career, Extracurricular research activities, Network building, Scientific pre-university program, Translational medicine

## Abstract

**Background:**

The proportion of medical doctors involved in research activities is declining. Undergraduate medical research programs are positively associated with medical students’ research interest. Scientific pre-university programs (SPUPs) outside the medical domain are also positively associated with research interest, but have not been related to the shortage of clinician-scientists. This study examined the effect of an SPUP on medical students’ research interest.

**Methods:**

This study was conducted at a Dutch medical school. Medical students in all years who had participated in an SPUP and first-year master students who had not participated in an SPUP were invited to fill out an online survey on extracurricular activities and future career interests. SPUP participants were compared with three groups of non-participants: (i) an unmatched group, (ii) a group matched on gender and pre-university Grade Point Average (pu-GPA) and (iii) a group matched on gender and first-year GPA, one to five years after finishing the SPUP. Participants evaluated the SPUP through ratings of statements about the program.

**Results:**

Two-hundred forty medical students, including 71 SPUP participants responded to the survey. SPUP participants participated significantly more often in the Honors class (i.e., extracurricular educational program for high-performing students), gained significantly more often extracurricular research experience, enrolled significantly more often in the Research master (i.e., research training program parallel to the clinical master program) and obtained significantly more often a scholarship than unmatched non-SPUP participants. Using a non-SPUP group matched on gender and pu-GPA reduced the effect size of the significant differences in Honors class participation, Research master participation and scholarship obtainment. Using a non-SPUP group matched on gender and first-year GPA rendered the significant difference in Research master participation and scholarship obtainment insignificant. Significantly more SPUP participants than unmatched non-SPUP participants preferred a combination of clinical care and research in their future career. Using a non-SPUP group matched on gender and either pu-GPA or first-year GPA did not change the effect size of this significant difference.

**Conclusions:**

These findings demonstrate the potential value of an SPUP in increasing the number of medical students with research interest and as a policy measure to help to alleviate the shortage of clinician-scientists.

**Electronic supplementary material:**

The online version of this article (10.1186/s12909-017-0990-4) contains supplementary material, which is available to authorized users.

## Background

The number of clinician-scientists has declined over the last decades [1, 2]. Between 1980 and 1997, the number of U.S. physicians who reported research as their primary activity decreased by 6%, while in the same period the number of physicians that indicated patient care as their primary activity almost doubled [3]. More recent figures show that during the last decade the total number of physicians increased, while the number of clinician-scientists remained unchanged [4]. This decline is often explained by the lack of research training in medical school and cuts in research budgets [5] and is alarming since the combination of patient care with research is essential for the translation of medical research into therapeutic applications [6]. It is therefore crucial to examine interventions aimed at stopping the decrease in the number of clinician-scientists.

Previous studies on the effects of research training *during* medical school demonstrated a positive association with students’ research interest [7, 8], interest in an academic career [7–9] and research productivity [10, 11]. However, research training could also be introduced *before* the start of medical school in the form of a scientific pre-university program (SPUP). SPUPs may add value over research training during medical school, because SPUPs provide an opportunity to gain authentic hands-on research experience which might enhance the stimulating effect of research training during medical school. Previous studies on SPUPs outside the medical domain indicated a positive impact of these programs on participants’ research interest. A large retrospective study, for example, showed that secondary school students who participated in a summer science program between 1958 and 1972 more often pursued a career in science compared to secondary school students whose first research experience took place during university [12]. Another study showed that excelling secondary school students who participated in a summer science program gained increased interest in a career in science [13, 14]. Extracurricular scientific activities, such as excursions, practical science work and guest lectures, stimulated secondary school students’ motivation to continue their participation in science [15]. Secondary school students who were randomly selected for a science exploration program showed a more positive attitude towards science and a higher interest in an academic career than classmates who were not selected for the program [16]. Luehmann [17] identified a number of important benefits of science enrichment programs in the literature on out-of-school programs, among others the increase in interest and motivation in science.

The above studies indicate that introducing secondary school students with scientific research before medical school might increase their motivation and interest in an academic career in medicine. The few studies on pre-medical school programs mainly focused on increasing the number of underrepresented minorities in the medical student population [18–20]. We are not aware of any studies relating pre-medical school science programs to the shortage of clinician-scientists. Therefore, the aim of this study was to examine the relationship between participation in an SPUP and medical students’ interest in research. It was expected that participation in an SPUP would be positively related to medical students’ research interest. Like previous research, this study also addressed participants’ appraisal of an SPUP. This study will provide information on the effect of an SPUP into a European setting. The majority of the above studies have been conducted in the U.S., while the decline in clinician-scientists is not limited to this area. Medical students with a greater interest in research might become medical doctors who are more willing to combine patient care with scientific research. Positive results would demonstrate the potential usefulness of an SPUP as an additional strategy to stop the decline in the number of clinician-scientists.

## Methods

### Context

This study was performed on an SPUP at the Erasmus MC Medical School, the Netherlands. The medical curriculum of the Erasmus MC Medical School takes six years and comprises a three-year (pre-clinical) bachelor degree and a three-year master degree (predominately clinical). Research training during the bachelor degree program is limited: literature reviews (e.g., journal clubs; year 1–3) and several scientific writing assignments of increasing difficulty (i.e., abstract (year 1), argumentation (year 2) and essay (year 3)). Later, in the master degree program students come into intensive contact with research during a 21-week research internship at a department of the student’s choice.

### SPUP - Junior Med School

**Fig. 1:**
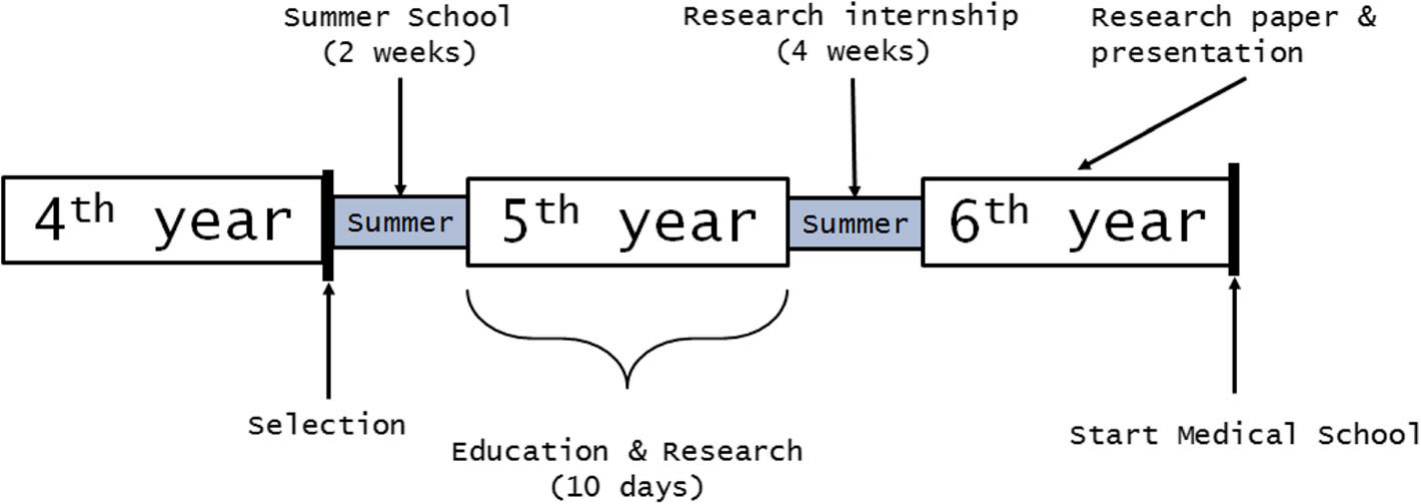
The Erasmus MC Junior Med School program across the last three years of pre-university education

In 2006, the Erasmus MC Medical School founded the Junior Med School (JMS) to provide a group of secondary school students the opportunity to gain research experience prior to medical school. The JMS is an SPUP that takes place during the last two years of secondary school at a pre-university education level (Fig. 1). The JMS was developed by a group of faculty members involved in teaching in all years of the medical curriculum. The aim of the program was to introduce the students to the methods of scientific research, to present various types of research subjects and projects within the medical context, and to give the students a hands-on experience with the actual practice of scientific research (i.e., preparation, the study itself, writing a report and present the results). Each year, a group of faculty members selects 24 excellent secondary school students for this program based on the students’ grades, résumé and performance during a series of short interviews framed as a Multiple Mini Interview [21]. The SPUP consists of four components: i) Summer School of two weeks duration, ii) ten days of education and research, iii) four-week research internship and iv) the writing and presentation of a research paper. The total program consists of 320 contact hours and takes place at the Erasmus MC campus. The Summer School takes place in the first summer of the program and consists of classes and practical courses. During the Summer School, several medical topics are considered, including the heart (Cardiology) and the brain (Neurosciences). Special attention is given to the scientific aspect of these topics. The ten days of education and research are spread across the first year of the program and consist of five pairs of two days devoted to five specific medical topics, such as transplantation and medical imaging. During the second summer of the program, students conduct a four-week research project in pairs. Conducting this project in pairs and in a laboratory setting offers students the possibility to gain hands-on experience with scientific research in a naturalistic environment. Students subsequently write a paper about their research projects and present it to Erasmus MC Medical School faculty members, fellow SPUP students and other invitees. Students who successfully pass the SPUP are granted direct access to the Erasmus MC Medical School. Since the foundation of the SPUP, 130 of the 143 participants (91%) have started at the Erasmus MC Medical School. Of the 13 SPUP participants that did not enter medical school, 11 pursued a biomedical science degree.

### Participants

The SPUP group of the present study consisted of former JMS participants who entered the Erasmus MC Medical School between 2009 and 2014. The 14 JMS participants who entered medical school in 2008 were excluded from this study, due to the experimental nature of this pilot year. The non-SPUP group consisted of 306 medical students enrolled in the first year of the master degree. The survey was administered before the students started the 21-week research internship. First-year master students instead of bachelor students were chosen, because it was expected that they would have had sufficient time and opportunity to participate in extracurricular activities. The survey was not administered to students in the second and third year of the master degree (i.e., clinical phase) because the clerkships in this period limit the time available for extracurricular activities. Moreover, this busy period makes it difficult to reach medical students for participating in surveys.

### Study design

This study involved a survey of extracurricular activities and future career interests, specially designed for this study. Between-group comparisons (SPUP vs. non-SPUP) were conducted of the answers to the survey. A common problem of between-group comparisons in this context is self-selection, which could bias the results due to pre-existing group differences. Such bias may be diminished by ensuring the comparability of the two groups on variables that are related to the outcome variable [22]. This study matched the SPUP and non-SPUP participants on gender and either pre-university Grade Point Average (pu-GPA) or first-year GPA, to investigate the possible effect of self-selection. We matched on gender because interest in scientific research may be associated with gender [23]. Since the JMS selects excelling secondary school students (i.e., selection bias), we matched on pu-GPA. The matching on first-year GPA was conducted because there may be an association between first-year GPA and certain extracurricular activities (e.g., Honors class).

### Procedure

The survey was administered online in the period of February and March 2015 and took on average ten minutes to complete. Students were invited to participate via email. In addition, the survey was announced during a lecture attended by first-year master students (i.e., the control group). To ensure that participants were blind for the true purpose of this study (i.e., comparison of SPUP vs. non-SPUP participants), the survey was introduced as an assessment of extracurricular activities. Participation in this study was voluntarily. Participants gave informed consent before they were navigated to the survey.

### Outcome measure

The survey started with demographic questions concerning gender, ethnicity and socio-economic status. Socio-economic status was represented by the level of education of the participant’s parents (first-generation university student) and whether one of the participant’s parents was appointed as a medical doctor. These data were used to examine the demographic comparability of the groups. The demographic questions were followed by a list of extracurricular activities, as shown in Table 1. Respondents indicated whether they had participated or were currently participating in these activities. The survey contained questions on extracurricular activities that were research related (e.g., published research paper(s), awarded scholarships) and non-research - related (e.g., additional credits obtained in other courses, special internships). Respondents were also asked about their participation in activities outside their studies (e.g., jobs, sports). When respondents indicated that they participated in a certain activity, additional questions were asked to enlighten the nature of these activities. The last part of the survey concerned the respondents’ interest concerning their future career (i.e., clinical care, research or a combination of clinical care and research). The survey consisted of at least 32 questions, depending on the answers given by the respondents. In addition, SPUP participants were asked to indicate how much they agreed with 13 statements about the SPUP using a five-point Likert scale (1: *Strongly disagree* – 5: *Strongly agree*). A copy of the survey is available [see Additional file 1].

**Table 1:** Short descriptions of the list of the extracurricular activities in the survey

Extracurricular activity	Description
*Non-research-related*	
Honors class	Educational program for excelling students in the first and second year of the bachelor degree
First-year clinical elective	Short internship in clinical health care during the first year of medical school
Anatomy program	Voluntary educational program focused on additional experience in the dissecting room
Tropical medicine course	Internship in a developing country
Education committee	Committee focused on the improvement of the quality of education
University council	Council representing students in the university board involved in policy decisions
Student year representation	Student group involved in the evaluation of the quality of education
Student hospital job	Part-time job in the hospital for medical students who obtained all credits of the first year
Clerkship council	Student board promoting the interest of medical students in their clerkships
Student association member	Member of the student association of the medical faculty
Committee student association	Member of a student committee involved in a specific topic (e.g., the yearbook)
Board student association	Student board promoting the interest of members of the student association
Credits in other programs	Obtainment of additional credits in courses outside medical school
Clerkship abroad	Clinical experience in a hospital outside the Netherlands
Clerkship other	Any other clinical experience
*Research-related*	
Extracurricular research experience	Activities such as data collection, entry and processing and laboratory work
Research master	Two-year research program parallel to and independent of the medical school master program
Authorship scientific paper	Author or co-author on a paper published in a scientific journal
Scholarship	Obtainment of financial aid
PhD	Started a PhD project

### Statistical analysis

Chi-squared tests were used to test whether the two groups differed in their participation in the extracurricular activities and their interest in a research career. Three between-group comparisons were conducted: between SPUP participants and i) an unmatched group of non-SPUP participants, ii) a group of non-SPUP participants matched on gender and pre-university Grade Point Average (pu-GPA) and iii) a group of non-SPUP participants matched on gender and first-year GPA. To ensure the comparability of the sample sizes, we selected non-SPUP groups with a similar sample size as the SPUP group. Because the survey was only administered to non-SPUP participants in the first year of the master degree, only SPUP participants in the master degree were used for comparison. Matching was preceded by categorizing pu-GPA and first-year GPA into nine categories (i.e., < 5.9 out of 10 = category 1, 6.0–6.4 = category 2, 6.5–6.9 = category 3 until 9.5 > = category 9). Next, for each participant in the SPUP group, we selected a participant from the non-SPUP group belonging to the same gender and pu-GPA or first-year GPA category. Last, we checked for differences in GPA and gender between the SPUP group and the matched non-SPUP group to determine if matching had succeeded. The effect size of the possible group differences is indicated with the phi coefficient.

The analysis of the evaluative statements involved data of all SPUP participants (i.e., bachelor and master degree). One-sample *t*-tests were performed to determine whether the statements about the JMS program significantly deviated from a rating of 4, which corresponds to “I agree with this statement”. A stricter alpha level was used (*α* < .01), due to the explorative nature of these 13 one-sample *t*-tests. Cohen’s *d* was used to determine the effect size of possible significant differences.

## Results

### Demographic characteristics

In total, 240 students (response rate = 58.1%) responded to the survey. Of this total, 71 respondents were former SPUP participants (response rate = 61.2%) and 169 were non-SPUP participants (response rate = 55.4%). SPUP participants were in their first (19.7%), second (11.3%) and third (22.5%) year of the bachelor degree, and in their first (18.3%), second (19.7%) and third (8.5%) year of the master degree. All non-SPUP participants were in their first year of the master degree. The demographic characteristics of the two groups are described in Table 2. SPUP participants were significantly younger (*t*(243) = −6.64, *p* < .001, *d* = 0.93), had a significantly higher pu-GPA (*t*(209) = 9.32, *p* < .001, *d* = 1.46) and a significantly higher first-year GPA (*t*(218) = 6.15, *p* < .001, *d* = 0.95). The two groups were comparable with regard to gender, ethnicity and socio-economic status.

**Table 2:** Descriptive statistics of the demographic characteristics of the SPUP and non-SPUP group

	SPUP (*N* = 71)	non-SPUP (*N* = 169)
Gender (female)	48 (67.6%)	115 (68%)
Age (Mean (SD))	**21.6** (1.8)	**23.6** (2.2)
Ethnicity (Dutch)	59 (83.1%)	129 (76.3%)
SES (first generation university students)	15 (21.1%)	50 (29.6%)
Medical docter as a parent	6 (8.5%)	22 (13%)
pu-GPA (Mean (SD))	**7.9** (0.5)	**7.1** (0.6)
first-year GPA (Mean (SD))	**7.1** (0.6)	**6.4** (0.7)

Bold numbers indicate a significant difference between the SPUP and non-SPUP group

*SPUP* Scientific Pre-university Program, *SD* Standard Deviation, *SES* Socioeconomic Status, *pu*-*GPA* pre-university Grade Point Average

### Extracurricular activities and career interest

These results involve only master students. For the non-research-related extracurricular activities, the SPUP group (*N* = 33) was significantly more often enrolled in the Honors class than the unmatched non-SPUP group (*N* = 33; *X* ^*2*^ (1) = 12.00, *p* = .001, φ = 0.43; Table 3). Honors class participation in the SPUP group was also significantly higher than in the non-SPUP group matched on gender and pu-GPA (*N* = 33; *X* ^*2*^ (1) = 4.99, *p* = .026, φ = .27) and the non-SPUP group matched on gender and first-year GPA (*N* = 32; *X* ^*2*^ (1) = 4.00, *p* = .046, φ = .25), although the effect size decreased from a relatively strong effect to a moderate effect. No significant difference was found in the obtainment of additional credits outside medical school between the SPUP group and the unmatched non-SPUP group. However, the SPUP group obtained significantly more often additional credits in courses outside medical school than the non-SPUP group matched on gender and pu-GPA (*X* ^*2*^ (1) = 4.69, *p* = .030, φ = .27) and the non-SPUP group matched on gender and first-year GPA (*X* ^*2*^ (1) = 4.45, *p* = .035, φ = .26), both with moderate effect sizes.

**Table 3:** Percentage of SPUP and non-SPUP participants that participated in the different extracurricular activities

		non-SPUP		
Extracurricular activity	SPUP (*N* = 33)	Not matched (*N* = 33)	Matched on pu-GPA (*N* = 33)	Matched on GPA year 1 (*N* = 32)
*Non-research-related*				
Honors class	42.4	**6.3**	**18.2**	**20**
First-year clinical elective	27.3	31.3	30.3	30
Anatomy program	27.3	18.2	12.1	20.7
Tropical medicine course	3	6.5	6.1	13.8
Education committee	6.1	0	6.1	10.3
University council	6.1	0	0	0
Student year representation	24.2	12.9	12.1	10.7
Student hospital job	75.8	63.6	54.5	56.3
Clerkship council	6.1	0	0	0
Student association member	78.8	90.9	72.7	87.5
Committee student association	54.5	60.6	63.6	65.6
Board student association	18.2	9.1	18.2	9.4
Credits in other programs	30.3	15.2	**9.1**	**9.4**
Clerkship abroad	6.1	0	0	0
Clerkship other	15.2	6.1	30.3	12.5
*Research-related*				
Extracurricular research experience	72.7	**36.4**	**36.4**	**31.3**
Research master	36.4	**3**	**15.2**	21.9
Authorship scientific paper	30.3	9.1	18.2	18.7
Scholarship	21.2	**0**	**3**	6.3
PhD	3	3	6.1	6.3
*Other activities*				
Jobs	81.8	90.9	81.8	84.4
Volunteering	18.2	27.3	27.3	28.1
Sports	66.7	84.8	81.8	71.9
Music	27.3	15.2	24.2	15.6
*Future*				
PhD	51.5	32.3	27.3	24.1
Interest - Clinic	6.1	**54.5**	**62.5**	**62.5**
Interest - Research	0	0	3	0
Interest – Clinic & Research	90.9	**39.4**	**34.4**	**34.4**

Non-SPUP participants were either not-matched, matched on gender and pu-GPA or matched on gender and first-year GPA with the SPUP participants. Bold percentages represent a significant difference between the SPUP and non-SPUP group, *p* < .05 (two-tailed)

*SPUP* = Scientific Pre-university Program, *pu*-*GPA* = pre-university Grade Point Average

Concerning research-related extracurricular activities, the SPUP group obtained significantly more often extracurricular research experience than the unmatched non-SPUP group, with a moderate effect size (*X* ^*2*^ (1) = 8.80, *p* = .003, φ = .37; Table 3). Significant differences in extracurricular research experience with similar effect sizes were found when the SPUP group was compared to the non-SPUP group matched on gender and pu-GPA (*X* ^*2*^ (1) = 8.80, *p* = .003, φ = .37) and the non-SPUP group matched on gender and first-year GPA (*X* ^*2*^ (1) = 11.20, *p* = .001, φ = .42). The SPUP group enrolled significantly more often in a Research master (i.e., research training program parallel to the clinical master program) than the unmatched non-SPUP group (*X* ^*2*^ (1) = 11.59, *p* = .001, φ = .42). Research master enrollment in the SPUP group was also significantly higher than in the non-SPUP group matched on gender and pu-GPA, but the effect size changed from relatively large to moderate (*X* ^*2*^ (1) = 3.88, *p* = .049, φ = .24). The difference in Research master enrollment was not significant between the SPUP group and the non-SPUP group matched on gender and first-year GPA (*X* ^*2*^ (1) = 1.65, *p* = .199). The SPUP group was significantly more often awarded with a scholarship than the unmatched non-SPUP group (*X* ^*2*^ (1) = 7.83, *p* = .005, φ = .34). The SPUP group also received significantly more scholarships than the non-SPUP group matched on gender and pu-GPA, but the effect size slightly reduced (*X* ^*2*^ (1) = 5.12, *p* = .024, φ = .28). Scholarship obtainment was not significantly different between the SPUP group and the non-SPUP group matched on gender and first-year GPA (*X* ^*2*^ (1) = 3.06, *p* = .081). No significant differences between the SPUP group and the non-SPUP groups, matched or not, were found for other activities, such as jobs, volunteering, sports and music.

Finally, a significant difference with a large effect size was found between the SPUP group and the unmatched non-SPUP group for the main focus of interest for students’ future careers (*X* ^*2*^ (2) = 21.51, *p* < .001, φ = .67; Table 3). Significant differences with similar effect sizes were found when the SPUP group was compared to the non-SPUP group matched on gender and pu-GPA and to the non-SPUP group matched on gender and first-year GPA (both *X* ^*2*^ (2) = 24.53, *p* < .001, φ = .61). A clear majority of the SPUP group indicated an interest in combining clinical care with research activities in their future career. This was significantly larger than the unmatched and matched non-SPUP groups of whom the majority indicated that they want to focus solely on clinical activities. An exclusive interest in research activities was only reported by a small proportion of the non-SPUP group matched on gender and pu-GPA.

To summarize, the most prominent and stable differences between the SPUP group and non-SPUP group were found for Honors class participation, extracurricular research experience and a future career interest in a combination of clinical care and research, all in favor of the SPUP group.

### Evaluation of the SPUP

**Fig. 2:**
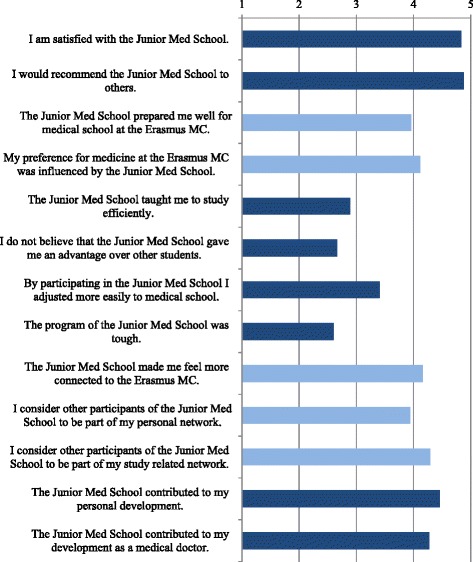
Evaluation of the JMS. Dark-colored bars indicate a significant deviation from a rating of 4 (Agree)

SPUP participants gave ratings significantly exceeding a rating of 4, indicating that they *agreed* to *strongly agreed,* for the following statements ‘I am satisfied with the Junior Med School’ (*t*(69) = 18.26, *p* < .001, *d* = 2.18, large effect), ‘I would recommend the Junior Med School to others’ (*t*(68) = 22.77, *p* < .001, *d* = 2.74, very large effect), ‘I consider other participants of the Junior Med School to be part of my study-related network’ (*t*(69) = 3.60, *p* = .001, *d* = 0.42, small effect),‘The Junior Med School contributed to my personal development’ (*t*(69) = 6.07, *p* < .001, *d* = 0.73, moderate effect), and ‘The Junior Med School contributed to my development as a medical doctor’ (*t*(69) = 3.07, *p* = .003, *d* = 0.37, small effect; Fig. 2). The following statements received evaluations that were significantly lower than a rating of 4, indicating that SPUP participants either disagreed with or were neutral regarding these statements: ‘The Junior Med School taught me to study efficiently’ (*t*(69) = −9.60, *p* < .001, *d* = 1.15, large effect), ‘I do not believe that the Junior Med School gave me an advantage over other students’ (*t*(69) = −11.17, *p* < .001, *d* = 1.34, very large effect), ‘By participating in the Junior Med School I adjusted more easily to Medical School’ (*t*(69) = −4.83, *p* < .001, *d* = 0.58, moderate effect) and ‘The program of the Junior Med School was tough’ (*t*(69) = −14.87, *p* < .001, *d* = 1.78, very large effect). The remaining 4 statements did not significantly differ from a rating of 4, implying that SPUP participants agreed with these statements (see Fig. 2).

To summarize, the SPUP received a positive evaluation with regard to overall appreciation, development as a person and medical doctor and network formation, but did not teach participants to study efficiently or helped them to adjust more easily to medical school.

## Discussion

This study found that participants of a scientific pre-university program (SPUP) were more involved in non-research and research-related extracurricular activities during medical school than non-SPUP participants. SPUP participants also indicated they were more willing to combine clinical care and research in their future career than non-SPUP participants. The significant difference in Research Master enrollment and scholarship obtainment disappeared due to matching on gender and first-year GPA, while the obtainment of additional credits in courses outside medical school emerged due to matching on gender and either pu-GPA or first-year GPA, indicating that matching on relevant variables can influence the differences between SPUP and non-SPUP participants. Finally, the SPUP program under study was positively evaluated by the participants.

SPUP participants were more involved in non-research-related extracurricular activities than non-SPUP participants (i.e., participation in the Honors class and obtaining additional credits outside the medical curriculum). No previous studies on SPUPs have addressed non-research-related extracurricular activities as an outcome variable. Although the larger participation in the Honors class might be explained by the higher academic performance of SPUP participants, matching on measures of academic performance did not cancel out the effect. The difference might be explained by certain characteristics of the SPUP participants, such as better planning skills. This hypothetical explanation is supported by a report on Career Academies [24], a secondary school program that provides small learning communities with a combination of academic and work-related courses. This report indicated that participants of the Career Academy are more often enrolled in a diverse mix of non-academic and vocational courses than non-participants. A difference in planning skills might also explain the significantly higher percentage of SPUP participants who obtained additional credit points from programs outside medical school. This significant difference was only present when the non-SPUP participants were matched on gender and pu-GPA or gender and first-year GPA. The study on the Career Academy showed that the enrollment in the extra non-academic courses did not come at the expense of completing academic courses [24]. Former SPUP participants might be more capable than non-SPUP participants in acquiring additional credit points, while at the same time keeping up their grades, thus possibly showing better planning skills. Future research should examine whether a difference in planning skills can actually explain these differences in non-research-related extracurricular activities and whether these skills are the result of participating in an SPUP or already existed before enrolling in an SPUP.

The higher participation rate in research-related extracurricular activities found among former participants of an SPUP demonstrates an association between research experience obtained prior to medical school and research interest during medical school. The participation rate among non-SPUP participants (36.4%) is comparable to the percentage of medical students who obtained extracurricular research experience (37.6%) reported in another Dutch study [11]. The greater involvement in research-related extracurricular activities among SPUP participants is in line with previous studies stating that participation in an SPUP increased student motivation to continue to participate in science [15] and to participate in additional extracurricular science programs [13]. The relationship between early research experience and continued research interest might be explained by the Social Cognitive Career Theory (SCCT) [25]. This theory states that the exposure to certain activities, such as research activities, positively influences self-efficacy concerning those activities and the anticipation of positive outcomes from those activities. Self-efficacy and outcome expectations are prominent factors in the development of interests. Thus, a greater involvement in research activities prior to medical school might increase self-efficacy and positive outcome expectations concerning research activities [25], which might strengthen students’ research interest during medical school. This continued research experience during medical school has also been demonstrated to be associated with a greater probability of a clinical research career [26, 27] and a higher scientific output [11].

The increased participation in research activities might persist even beyond medical school as shown by the significantly larger number of SPUP participants expressing a preference for a combination of patient care and research in their future career. This substantially higher interest corresponds with prior studies that concluded that an SPUP leads to a higher interest in a science career [16] and to a higher likelihood of actually pursuing a research career [12]. The attractive features of an SPUP program (i.e., more intensive course with like-minded peers) and the continuing cycle of the SCCT are possible explanations for the relationship between participation in an SPUP and a continued research interest even after graduating from medical school.

The participants of the SPUP positively evaluated the program, which is reflected by positive ratings on statements concerning network development and feeling connected to the Erasmus MC Medical School. The overall positive evaluation of the SPUP is in line with previous research which indicated that most students view research programs positively [7]. The most valuable aspect in the SPUP evaluation concerns the opportunity to form social networks. This corresponds to a previous study in which participants of a science enrichment program indicated that the program helped them in the development of networks or friendships with other participants [28]. The development of a network with other students is important for the involvement in the academic environment, which is an essential factor in academic performance [29].

This study has important implications for medical schools that aim to increase the number of clinician-scientists. Since most medical school programs have a limited amount of time available for research training in their curricula, SPUPs could offer a possible solution to increase research interest among medical students. In addition, medical schools should offer students the opportunity to participate in research-related extracurricular activities during medical school allowing students to expand the research experience they obtained prior to medical school. Further research is necessary on the generalizability of these findings from the Dutch setting to settings where medical school does not immediately follow secondary school. Furthermore, SPUPs may also be implemented as a resource for future medical students to form social and study-related networks, which may contribute to their academic performance.

This study is not without limitations. Firstly, although this study is one of the first that made an attempt to actively counteract the self-selection problem by matching SPUP participants and non-SPUP participants on gender and pu-GPA or gender and first-year GPA, self-selection on other variables might still influence the results. Especially, pre-existing differences in research interest and motivation may explain the difference in participation rate in research-related extracurricular activities. Future studies should include a baseline measurement of these variables to allow the study of their effect on self-selection. Secondly, the sample size of this study is small due to the low number of secondary school students that is selected for the SPUP under study. Nevertheless, interesting differences were detected, even when the groups were matched on gender and pu-GPA or gender and first-year GPA. Future research should, however, repeat this study with a larger sample size to replicate our findings and further unravel the effects of an SPUP on medical students’ research interest. Thirdly, the non-SPUP group consisted of only first-year master degree medical students because logistical reasons prevented the survey to be administered to all medical students. Future research should be conducted among medical students of all academic years to examine the effect of an SPUP over the course of the medical curriculum. First-year master degree students, however, provided a good comparison group because they had sufficient time to participate in extracurricular activities but were not preoccupied by the clerkships, unlike second-year and third-year master degree medical students. Fourthly, since none of the former SPUP participants has graduated yet, no conclusions can be drawn about their actual career choices. Although the large number of SPUP participants interested in the combination of clinical care with research seems promising, a longitudinal study is crucial to determine whether the greater interest in future research activities stated by the SPUP participants will translate to actual future research activities. The final limitation involves the retrospective evaluation of the SPUP. A survey immediately at the end of the SPUP could result in a more accurate evaluation.

## Conclusions

In conclusion, former SPUP participants enrolled significantly more often in the Honors class, gained significantly more extracurricular research experience, enrolled significantly more often in the Research master and were significantly more often awarded with a scholarship than non-SPUP participants. In addition, significantly more SPUP participants than non-SPUP participants indicated an interest in the combination of clinical care and research in their future career. Only the differences in Honor class participation, extracurricular research experience and future career interest remained significant after matching on gender and either pu-GPA or first-year GPA, demonstrating the importance of taking into account pre-existing differences that could lead to self-selection bias. The SPUP program received a positive evaluation of the participants and helped the participants in the formation of a social network. The results suggest that a research program prior to medical school might positively impact research interest among medical students and could offer an additional solution to the shortage of clinician-scientists.

## Additional file

Additional file 1: Survey SPUP study. The survey that was administered to the participants of this study. (DOCX 29 kb)

## Abbreviations

GPA: Grade Point Average
JMS: Junior Med School
pu-GPA: pre-university GPA
SPUP: Scientific Pre-university Program
