# Supplementary material for: Participation in a scientific pre-university program and medical students’ interest in an academic career
Source: BMC Med Educ. 2017 Sep 5;17:150. doi: 10.1186/s12909-017-0990-4 (PMC5584329; doi:10.1186/s12909-017-0990-4)
Supplement: Additional file 1: — Survey SPUP study. The survey that was administered to the participants of this study. (DOCX 29 kb) [file 12909_2017_990_MOESM1_ESM.docx]

**Survey SPUP Study**

**Demographics**

1. What is your student number?

2. In which academic year are you?

- 1
- 2
- 3
- 4
- 5
- 6
- 7 or higher
- Graduated

3. Did at least one of your parents / guardians complete an education at either a research university or a university of applied science?

- Yes
- No

4. Does at least one of your parents / guardians work as a medical doctor or did at least one of your parents / guardians work as a medical doctor?

- Yes
- No

5. Where were you born?

- In the Netherlands *(continue to question 7)*
- Outside the Netherlands, namely:

6. For how many years have you been living in the Netherlands?

7. Where was your father born?

- In the Netherlands
- Outside the Netherlands, namely:

8. Where was your mother born?

- In the Netherlands
- Outside the Netherlands, namely:

9. In what language do you communicate with your parents?

- Only Dutch
- Other, namely:

**Extracurricular activities**

10. Do you participate / have you participated in the following activities?

(Yes/No/Not applicable)

- Junior Med School (*no or not applicable 🡪 skip question 13*)
- Honors class
- First-year clinical elective
- Anatomy program
- Tropical medicine course
- Education committee
- University council
- Student year representation
- Student hospital job
- Clerkship council

11. Have you applied for the following extracurricular activities?

(Yes/No/Not applicable)

- Junior Med School
- Honors class
- First-year clinical elective
- Anatomy program
- Tropical medicine course
- Education committee
- University council
- Student year representation
- Student hospital job
- Clerkship council

13. You indicated that you participated in the Junior Med School. We would like to know how you have experienced the Junior Med School by presenting a number of statements to you. Indicate for each statement if you strongly disagree, disagree, neutral, agree or strongly agree.

- I am satisfied with the Junior Med School.
- I would recommend the Junior Med School to others.
- The Junior Med School prepared me well for medical school at the Erasmus MC.
- My preference for medicine at the Erasmus MC was influenced by the Junior Med School.
- The Junior Med School taught me to study efficiently.
- I do not believe that the Junior Med School gave me an advantage over other students.
- By participating in the Junior Med School I adjusted more easily to medical school.
- The program of the Junior Med School was tough.
- The Junior Med School made me feel more connected to the Erasmus MC.
- I consider other participants of the Junior Med School to be part of my personal network.
- I consider other participants of the Junior Med School to be part of my study related network.
- The Junior Med School contributed to my personal development.
- The Junior Med School contributed to my development as a medical doctor.

14. Are you / have you been a member of a student association?

- Yes
- No (*continue to question* *24*)

15. In which academic year / years were you a member of a student association?

- 1
- 2
- 3
- 4
- 5
- 6

16. Of which student association are / were you a member?

17. Do / did you participate in any committee of your student association?

- Yes
- No (*continue to question 20*)

18. In which academic year /years were you in a committee?

- 1
- 2
- 3
- 4
- 5
- 6

19. In which committee are you participating / did you participate?

20. Are you / have you been in the board of your student association?

- Yes
- No (*continue to question 24*)

21. In which academic year were you on the board of your student association?

- 1
- 2
- 3
- 4
- 5
- 6

22. At which student association are / were you in the board?

23. Which position are you fulfilling / did you fulfill in the board?

24. Did you obtain additional credits in other programs during medical school?

- Yes
- No

25. Which minor did you follow? (*only if question 2 is answered with 3 or higher*)

26. Did you follow any clerkships abroad?

- No (*continue to question 31*)
- Yes, a regular clerkship (*skip questions 28 and 30*)
- Yes, an elective clerkship (*skip questions 27 and 29*)
- Yes, a regular and an elective clerkship

27. In what country did you follow your regular clerkship?

28. In what country did you follow your elective clerkship?

29. At what kind of institution did you follow your regular clerkship?

- Academic hospital
- General hospital
- Private hospital

30. At what kind of institution did you follow your elective clerkship?

- Academic hospital
- General hospital
- Private hospital

31. Did you follow any special internships?

- Yes
- No (*continue to question 34*)

32. Where did you follow this / these special internship / internships?

33. In which academic year did you follow this / these special internship / internships?

- 1
- 2
- 3
- 4
- 5
- 6

34. Did you apply for the fast track program? (*only if question 2 is answered with 4 or higher*)

- Yes
- No *(continue to question 37)*
- Not applicable *(continue to question 37)*

35. At which department did you apply for the fast track program?

- Anesthesiology
- Cardiology
- Geriatrics
- Surgery
- Family medicine
- Internal medicine
- Pediatrics
- Ear, Nose, Throat
- Gastroenterology
- Orthopedics
- Pathology
- Psychiatry
- Radiology
- Gynecology and obstetrics

36. Have you been selected for the fast track program?

- Yes
- No
- Do not know yet

37. Did you gain any research experience outside the regular curriculum?

- Yes
- No (*continue to question 39*)

38. Please describe this research experience?

39. Do you participate / have you participated in the research master?

- No
- Yes, namely Health Science (*skip question 40*)
- Yes, namely Infection & Immunity (*skip question 40*)
- Yes, namely Neuroscience (*skip question 40*)
- Yes, namely Clinical Research (*skip question 40*)
- Yes, namely Molecular Medicine (*skip question 40*)
- Yes, namely MADE (European Master of Science) (*skip question 40*)

40. Are you planning to participate in the research master?

- No
- Yes, namely Health Science
- Yes, namely Infection & Immunity
- Yes, namely Neuroscience
- Yes, namely Clinical Research
- Yes, namely Molecular Medicine
- Yes, namely MADE (European Master of Science)
- Yes, but I do not yet know which research master
- Do not know yet

41. Are you an author on one or more scientific papers?

- No (*continue to question 48*)
- Yes, on 1 paper (*skip questions 43, 45 and 46*)
- Yes, on 2 papers (*skip questions 42, 44 and 46*)
- Yes, on 3 or more papers (*skip question 42, 44 and 45*)

42. At what author position are you on this paper?

43. At what author position are you on these papers?

44. Has this scientific paper been accepted / published?

- Yes
- No (*continue to question 48*)

45. Have these scientific papers been accepted / published?

- No (*continue to question 48*)
- Yes, 1 paper
- Yes, both papers

46. Have these scientific papers been accepted / published?

- No (*continue to question 48*)
- Yes, 1 paper
- Yes, 2 papers
- Yes, 3 or more papers

47. In which journal / journals did you publish this paper / these papers?

48. Have you been awarded by one or more scholarships?

- No
- Yes, namely:

49. Are you conducting a PhD project? (*only if question 2 is answered with 4 or higher*)

- Yes (*skip questions 52 and 53*)
- No (*continue to question 52*)

50. At which department are you conducting your PhD project?

51. What is the title of your PhD thesis?

52. Are you planning to conduct a PhD project?

- Yes
- No
- Do not know

53. At which department would you like to conduct your PhD project?

54. If you participated in any other extracurricular activities, please describe these activities below:

**Activities other than education**

55. Do you have a job (no volunteer work)?

- Yes
- No (*continue to question 58*)

56. What kind of job do you have?

57. How many hours per week on average do you spend on your job?

58. Do you do volunteer work?

- Yes
- No (*continue to question 61*)

59. What kind of volunteer work do you do?

60. How many hours per week on average do you spend volunteering?

61. Do you practice sports?

- Yes
- No (*continue to question 64*)

62. What kind of sports do you practice?

63. How many hours per week on average do you spend on practicing sports?

64. Do you play a musical instrument?

- Yes
- No (*continue to question 67*)

65. What musical instrument do you play?

66. How many hours per week on average do you play a musical instrument?

67. If you participate in any other activities outside your education, please state below:

**Future**

68. Do you want to enter specialty training after finishing medical school?

- Yes
- No (*continue to question 70*)
- Do not know (*continue to question 70*)

69. At which department would you like to do your specialty training?

- Anesthesiology
- Occupational medicine
- Insurance medicine
- Cardiology
- Cardiothoracic surgery
- Dermatology and venereology
- Medicine for the mentally disabled
- Surgery
- Family medicine
- Internal medicine
- Ears, Nose, Throat
- Pediatrics
- Clinical genetics
- Clinical geriatrics
- Pulmonary diseases and tuberculosis
- Gastroenterology
- Society and health
- Society and health 2^nd^ phase
- Medical microbiology
- Neurosurgery
- Neurology
- Nuclear medicine
- Obstetrics and gynecology
- Ophthalmology
- Orthopedics
- Pathology
- Plastic surgery
- Policy and advice
- Forensic medicine
- Immunology prevention
- Youth healthcare
- Environmental health
- Social medical assessment and consultancy
- Emergency medicine
- Tuberculosis prevention
- Addiction medicine
- Psychiatry
- Radiology
- Radiotherapy
- Rheumatology
- Rehabilitation medicine
- Geriatrics
- Urology
- Do not know yet

70. If you do not want to enter specialty training, what do you want to do after finishing medical school?

71. Where lies your main interest?

- Clinic
- Research
- Both
- Other, namely:

72. Any addition or remarks can be stated below:
